# Supplementary material for: The effect of nasal and oral breathing on airway collapsibility in patients with obstructive sleep apnea: Computational fluid dynamics analyses
Source: PLoS One. 2020 Apr 13;15(4):e0231262. doi: 10.1371/journal.pone.0231262 (PMC7153879; doi:10.1371/journal.pone.0231262)
Supplement: Attachment — Submitted filename: Response to Reviewers.docx [file pone.0231262.s001.docx]

Response to Reviewers

We would like to thank the reviewers for the latest review and apologize for delaying the resubmission of this revised manuscript. We have answered each of the points below. We increased the sample size and reorganized the manuscript to try to improve the logical flow of our argument. We hope that this more substantial reworking of the manuscript is satisfactory.

**Reviewer #1:**
Major comments:
Is there any way to identify or assess the anatomical features such as a position of tongue during three types of breathing? It is just curiosity of this reviewer.
Thank you for pointing this out. The STL models revealed that the tongue touched the hard palate during nasal breathing with closed mouth, whereas a low tongue position that did not touch the hard palate was observed during nasal and oral breathing with open mouth.

We have added these findings to the Results section.
The sample size is just 6 OSA patients, so suggestion would be that assessment of SDB characteristics and computational fluid dynamics in each patient should be performed if possible. Might be interesting.
Thank you for pointing this out. Statistical power test suggested that sample needs more than 12.14 under the conditions of delta; 0.3, sd; 0.2, sig.level; 0.01, and power; 0.8. Therefore, we added eight patients to make sample size 14. Significant difference was observed between the nasal breathing with closed and open mouth in the wall shear stress, however, other statistical comparisons showed almost the same results as before.

We changed the Result section.

Minor comment:
The authors looked at only inspiration phase. Did the authors instruct the patients how to exhale, i.e. breath out via mouth or nose, and so forth?

Thank you for pointing this out. During oral breathing, patients breathed 28.7% ± 3.3% via the nose and 71.3 ± 5.1% via the mouth during inspiration, and breathed 20.4% ± 2.5% via the nose and 79.6% ± 4.4% via the mouth during expiration (*p*= 0.17, 2x2 Chi square test). In contrast, patients breathed 100% via the nose both in inspiration and expiration during nasal breathing, even with open mouth.

We have added these findings and CFD figures during expiration (Fig. 4) to the Results section.

**Reviewer #2:**
1. The study topic is vague. What did you want to compare? The topic should include the examination condition (conscious status or sleep status?)

Thank you for pointing this out. The purpose of this study was to investigate the effect of breathing route on the collapsibility of the pharyngeal airway, represented by airflow velocity and static pressure calculated using CFD technology, in patients with OSA.

We changed the title, Introduction, and objective of the abstract to make this clear.
2. The conclusions of the abstract are not supported by the results (There was no result showing oral breathing increased pharyngeal collapse.)

Thank you for pointing this out. We elucidated the pharyngeal airway collapsibility based on the concept of the Starling Resistor model. Collapsibility is dependent on the airflow velocity and the static pressure through the oropharynx. Detailed values for airﬂow velocity and static pressure through the pharyngeal airway can be calculated using CFD. Our results showed that airflow velocity and static pressure were significantly increased during oral breathing, indicating that airflows with different velocities merged to generate friction and swirl, which led to loss of pressure and an increase in entropy that facilitated collapse.

We changed the Abstract and have added more detailed explanation of collapsibility to the Discussion section.

simply duplication (The airflow during nasal breathing with the closed mouth was smoother than that with open mouth.).

We have avoided the duplication in the Abstract.
3. In the method section, please illustrate the procedures to control the respiratory force during nasal breathing without the mouth opening, nasal breathing with the mouth opening, and mouth breathing? If the respiratory forces were significantly different, measurements of CFD parameters (such as velocity) might be not objective.

Thank you for pointing this out. We measured volumetric flow rates in a steady breathing state as a substitute marker for ventilatory drive using a pneumotachometer along with a pressure sensor rather than performing diaphragm electromyography during nasal breathing with closed mouth, nasal breathing with open mouth, and oral breathing. Simulation models were confirmed to agree with these measured values.

We have added this explanation to the Methods.

4. Please provide several tables to summarize your results because the readers cannot track your data according to the figures.

Thank you for pointing this out. We have added a summarized table of CFD results (Table 1).
5. How did you decide your sample size? Did you evaluate the data distribution (normal or not normal?) and the statistical power? Because the sample size of this study was relatively small, this study was very preliminary and hard to make a conclusion.

Thank you for pointing this out. Statistical power test suggested that sample needs more than 12.14 under the conditions of delta; 0.3, sd; 0.2, sig.level; 0.01, and power; 0.8. Therefore, we added eight patients to make sample size 14. Significant difference was observed between the nasal breathing with closed and open mouth in the wall shear stress, however, other statistical comparisons showed almost the same results as before.

We changed the Result section.

6. The discussion section was not well-written and effective. The authors should compare their findings with other studies to explain specific research outcomes. The authors may explain the reasons for these findings are specific to OSA. Please consider writing a more effective discussion.

Thank you for pointing this out. We have reorganized the Discussion to try to improve the logical flow of our argument. We have added essential discussion concerning velocity, pressure and collapsibility to the Discussion section, and deleted non-effective sections.

7. During awake, conscious states, patients could breathe with different efforts resulting in a larger bias and did not mimic to sleep-disturbed breathing events. Please consider to add it to your limitation.

Thank you for pointing this out. CT scans were performed while patients were conscious; thus, patients could have breathed with different amounts of effort, resulting in a large bias.

We have added this limitation section in the Discussion.
8. Please request for English editing of this manuscript. There were many grammar errors and confusing sentences in the manuscript.

The paper has been checked by a native English-speaking medical editor.
